# Supplementary material for: Intestinal Explant Cultures from Gilthead Seabream (Sparus aurata, L.) Allowed the Determination of Mucosal Sensitivity to Bacterial Pathogens and the Impact of a Plant Protein Diet
Source: Int J Mol Sci. 2020 Oct 14;21(20):7584. doi: 10.3390/ijms21207584 (PMC7588912; doi:10.3390/ijms21207584)
Supplement: Supplementary file 1 [file ijms-21-07584-s001.pdf]

International Journal of *Molecular Sciences*

Article

Intestinal Explant Cultures from Gilthead Seabream (*Sparus aurata, L.)* Allowed the Determination of Mucosal Sensitivity to Bacterial Pathogens and the Impact of a Plant Protein Diet

David Sánchez Peñaranda [1,*,†]; Christine Bäuerl [2,†]; Ana Tomás-Vidal [1]; Miguel Jover-Cerdá [1]; Guillem Estruch [1]; Gaspar Pérez Martínez [2,‡] and Silvia Martínez Llorens [1,‡]

1 Aquaculture and Biodiversity Research Group, Institute of Science and Animal Technology (ICTA), Universitat Politècnica de València, 46022 Valencia, Spain; atomasv@dca.upv.es (A.T.-V.); mjover@dca.upv.es (M.J.-C.); guiescu@etsia.upv.es (G.E.); silmarll@dca.upv.es (S.M.L.)

2 Department of Biotechnology, Institute of Agrochemistry and Food Technology, Consejo Superior de Investigaciones Científicas (CSIC) (Spanish National Research Council), 46980 Paterna, Valencia, Spain; cbauerl@iata.csic.es (C.B.); gaspar.perez@iata.csic.es (G.P.M.)

* Correspondence: dasncpea@dca.upv.es; Tel.: +34-9638-79434

† These authors contribution equally to this work.

‡ These authors contribution equally to this work.

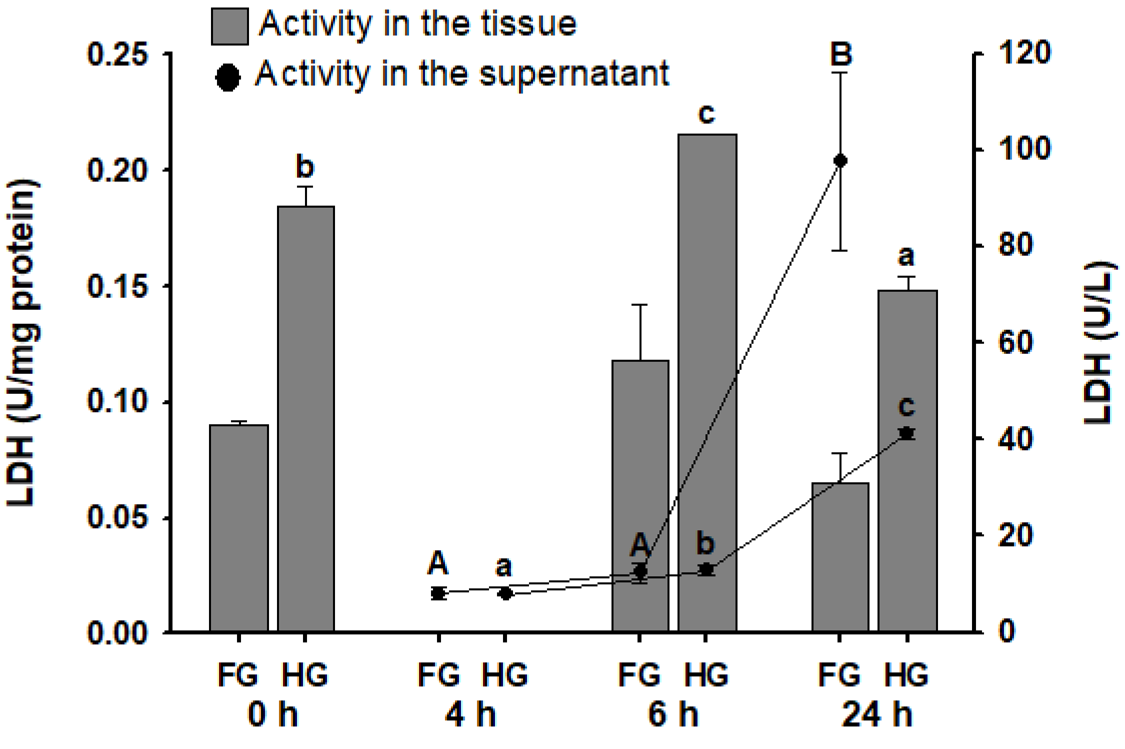

Supplementary Figure S1. Assessment of tissue integrity: Lactate dehydrogenase (LDH) activity. LDH activity in the tissue (U/mg protein) and in the DMEM supernatant (U/L) is expressed by the mean and standard error, for both intestinal sections (FG and HG) and four different times (0, 4, 6 and 24 h). Different superscripts on the bars indicate significant differences between different times for each section ($p < 0.05$). For supernatant comparison, capital letters indicate differences at foregut level, meanwhile lower case letters indicate differences at hindgut level.

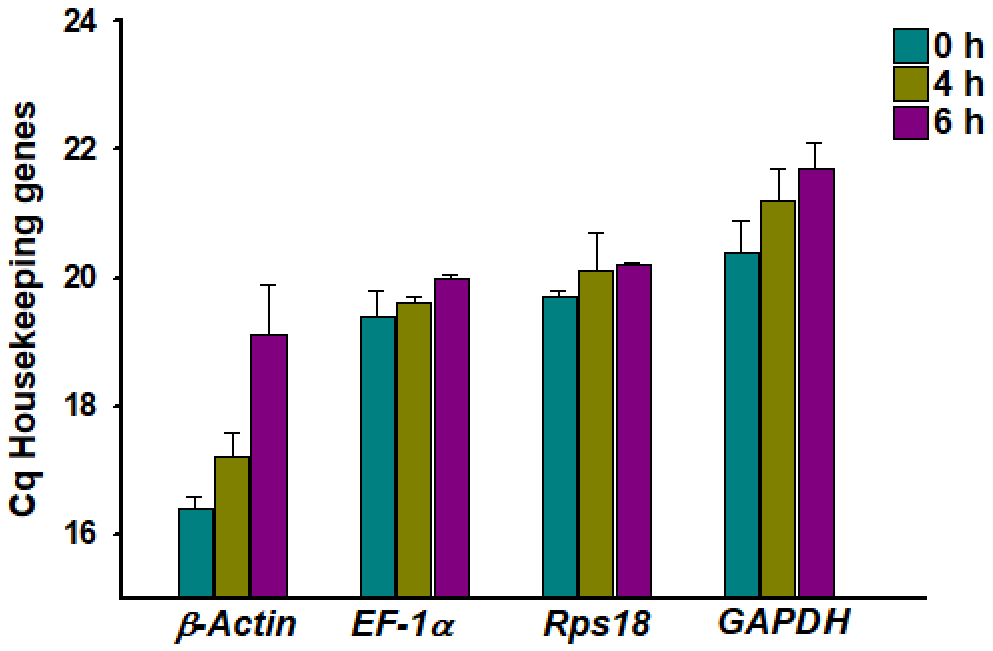

Supplementary Figure S2. Evaluation of the candidate housekeeping gene stability along the assay. Cq of the different genes in pooled samples at different times is expressed by mean and standard error. Different superscripts on the bars indicate significant differences between different times for each section ($p < 0.05$).

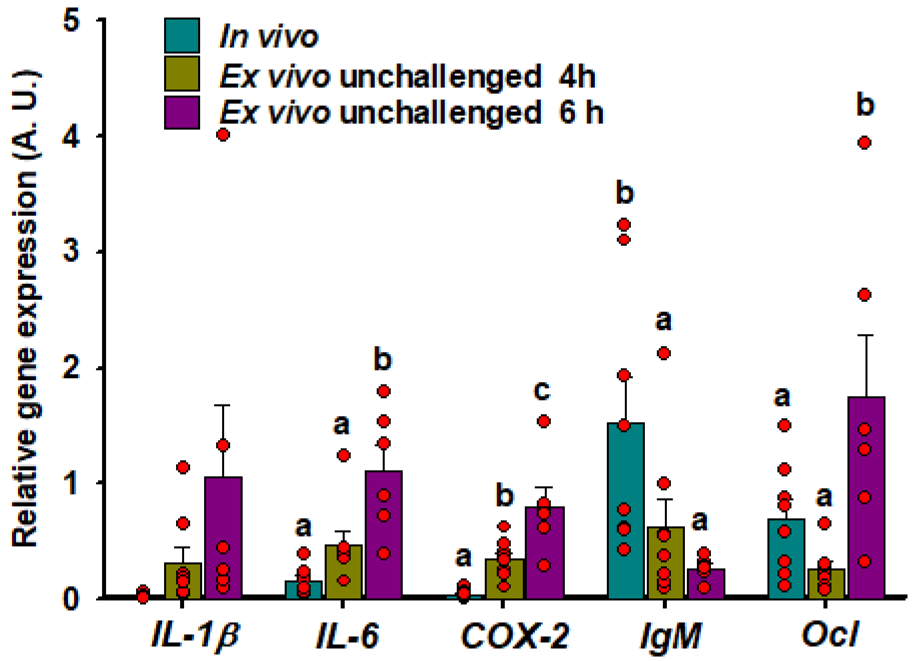

Supplementary Figure S3. Effect of ex vivo culture on the intestinal expression in Phase I (68 g). Relative gene expression (A. U.) of the different genes is expressed by the mean and standard error for the three different times (0, 4 and 6 hours). Different superscripts on the bars indicate significant differences between different times for each gene ($p < 0.05$). Data sample in each experimental group is represented by a red spot.

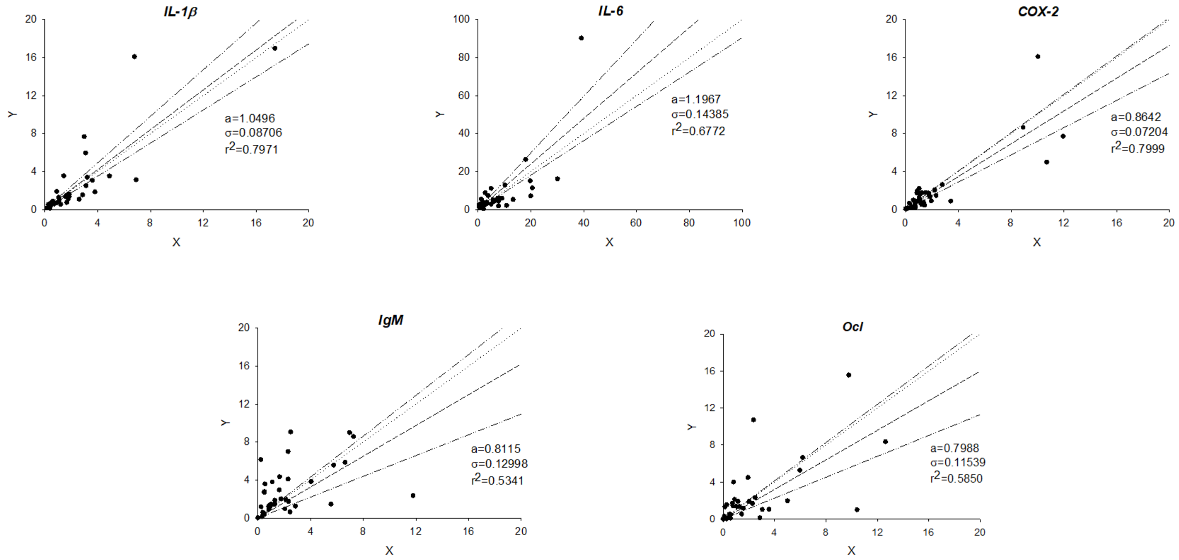

Supplementary Figure S4. Reproducibility assessment of the ex vivo assay. Relative gene expression values of duplicate samples for the same conditions (diet, section and stimuli) were represented as 'x,y'. The linear models y = *a*x and limits of the 95% confidence intervals were represented for each gene. The value *a* = 1 is included in the 95% confidence interval for all genes, then the hypothesis y = x cannot be rejected. Values for *a*, *σ* and *r2* are also included.

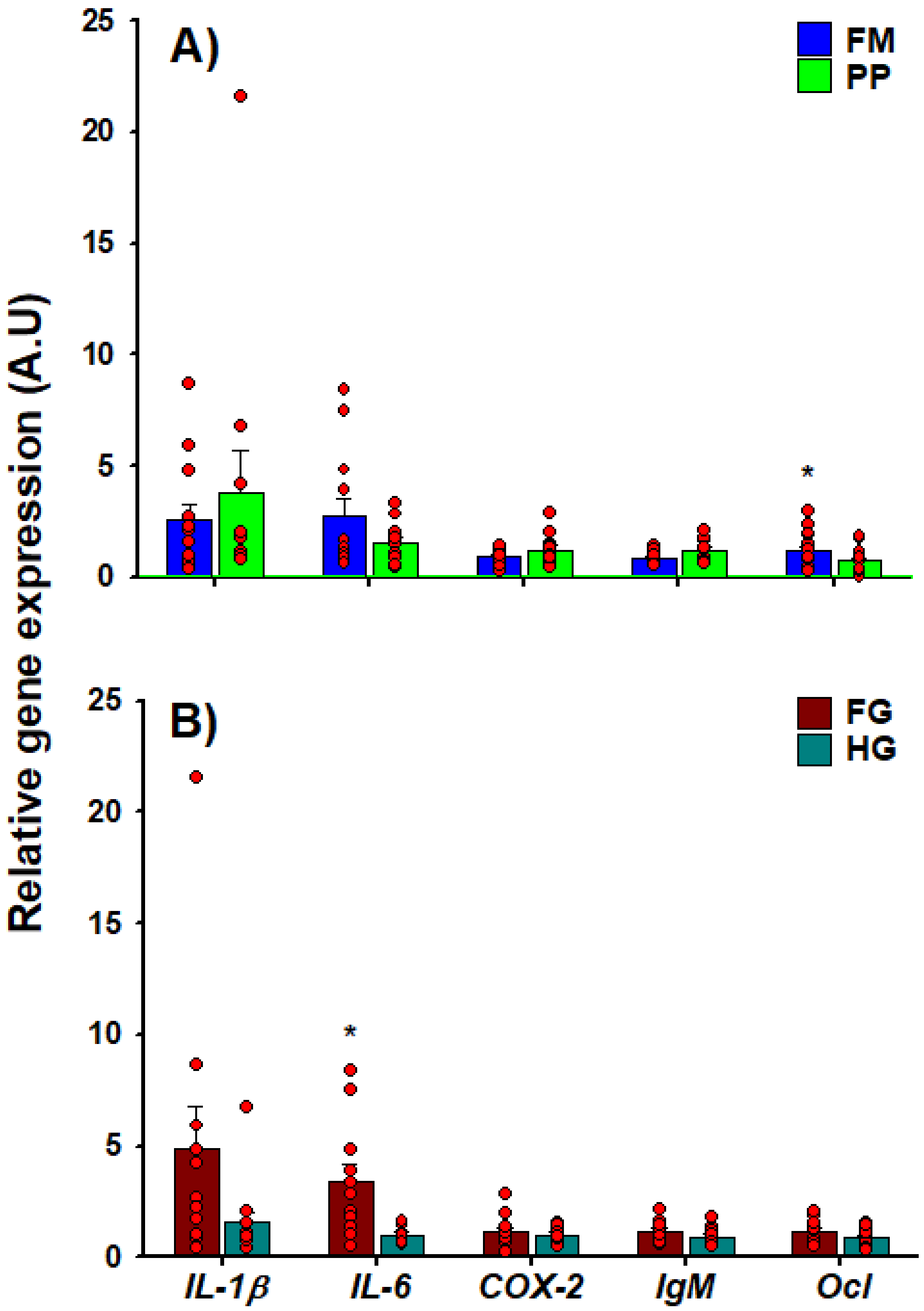

Supplementary Figure S5. Effect of diet and intestinal section after 4h of ex vivo bacterial exposition in Phase I (68 g). Relative gene expression (A. U.) of the different genes is expressed by the mean and standard error. Asterisk on the bars indicate significant differences between different conditions (diet/section) for each gene ($p < 0.05$) at 4h of incubation. A) Effect of dietary treatment B) Effect of intestinal section. Data sample in each experimental group is represented by a red spot.

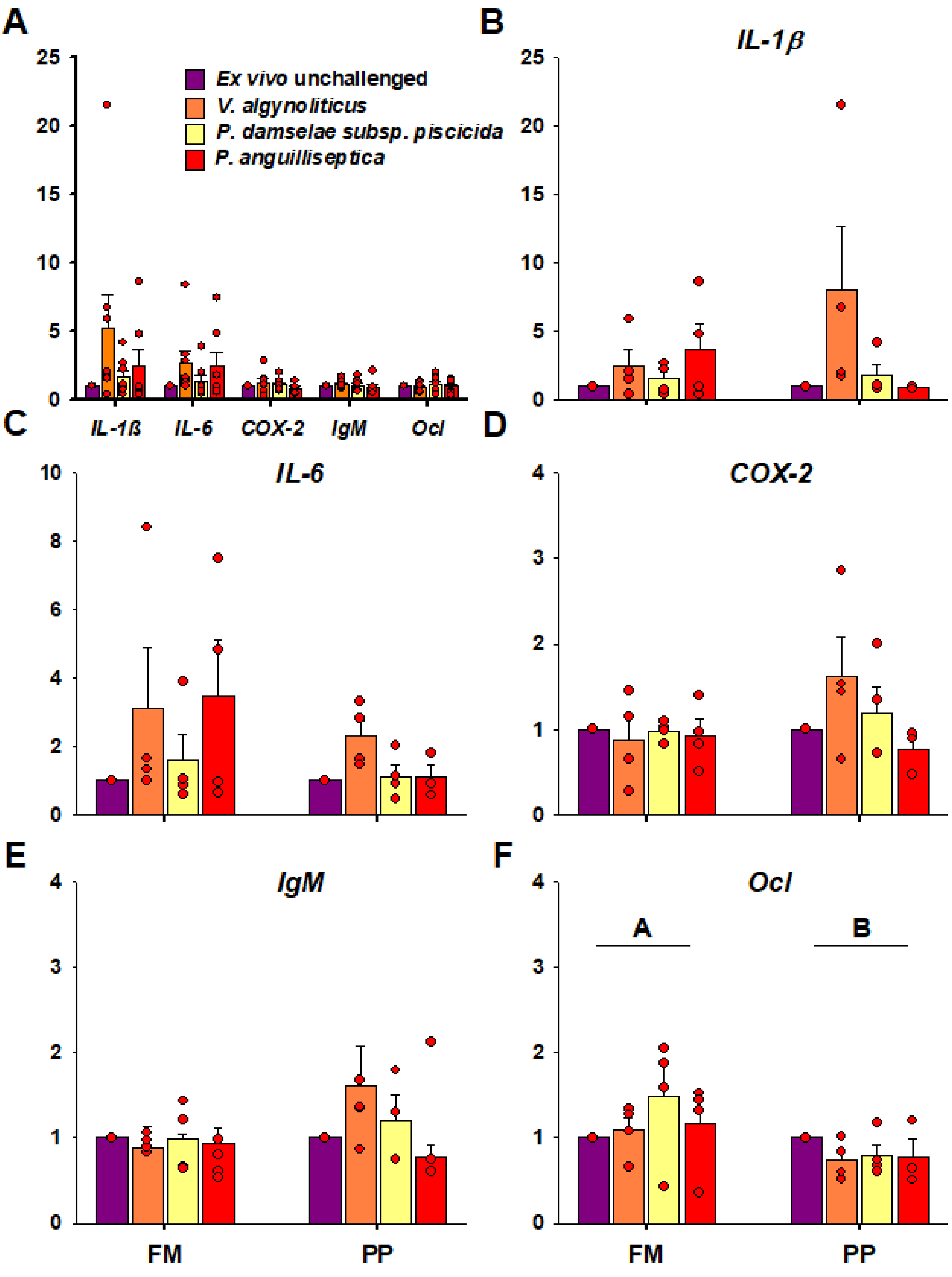

Supplementary Figure S6. Effect of bacterial challenge based on the diet after 4h of ex vivo bacterial exposition in Phase I (68 g). Capital letters indicate differences between diets, meanwhile lower case letters indicate differences between bacterial for each gene ($p < 0.05$). A) Effect of bacterial challenge independently of diet. Gene expression expressed by the mean and standard error of B) *IL-1β* C) *IL-6* D) *COX-2* E) *IgM* F) *Ocl* based on the stimuli and diet. Data sample in each experimental group is represented by a red spot.

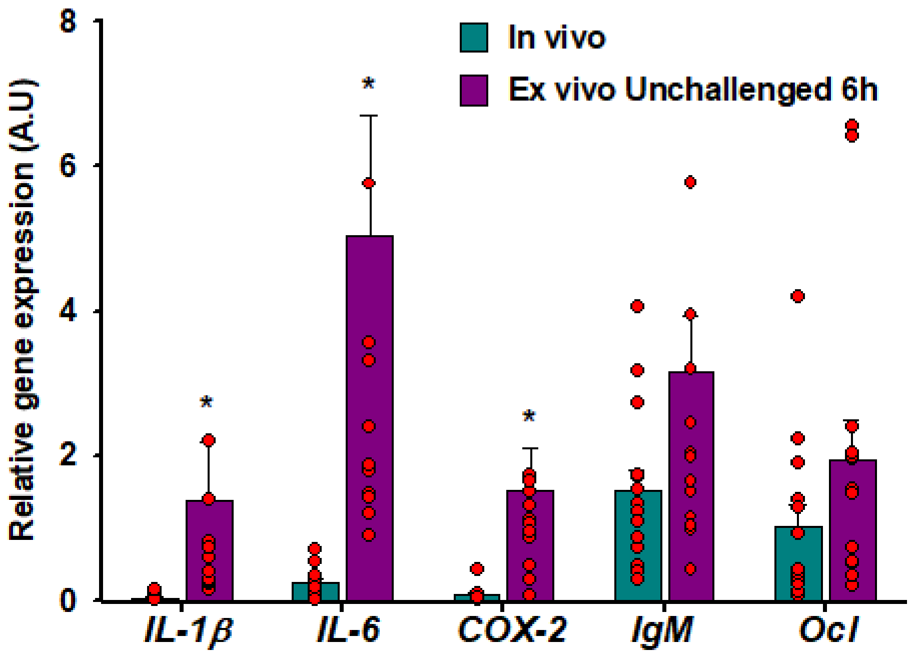

Supplementary Figure S7. Impact of ex vivo culture at Phase II (250 g). Relative gene expression (A. U.) of the different genes is expressed by the mean and standard error. Asterisks on the bars indicate significant differences for each gene ($p < 0.05$) after 6 hours of incubation. Data sample in each experimental group is represented by a red spot.

Table 1. Effect of different factors on normalised gene expression values in Bacterial Challenged Phase I (68 g).

		IL-1β	*IL-6*	*COX-2*	*IgM*	*Ocl*
**At 4 h**	*Diet*	0.481	0.181	0.228	0.074	0.017 *
	Section	0.078	0.005 *	0.670	0.223	0.098
	Stimuli	0.145	0.116	0.523	0.771	0.680
**At 6 h**	*Diet*	0.074	0.011 *	0.011 *	0.510	0.007 *
	Section	0.391	0.336	0.133	0.516	0.015 *
	Stimuli	0.001*	0.083	0.024 *	0.137	0.101

p-values obtained for each factor in the multifactorial analysis. Significant values are indicated by *.*Interleukin 1β: IL-1β; Interleukin 6:I L-6; Cyclooxygenase 2: COX-2; Immunoglobulin M: IgM; Occludin: Ocl*
